# Biological Control of *Microcystis aeruginosa* Through Sequestration in Pseudofaeces Produced by the Freshwater Gastropod, *Sinotaia aeruginosa*

**DOI:** 10.3390/toxins17110536

**Published:** 2025-10-30

**Authors:** Barry N. Madison, Mingzhi Qu, Elliot Gavrin, Wenwei Ren, Yuxiang Wang, Daniel D. Lefebvre

**Affiliations:** 1 Department of Biology, Brandon University, Brandon, MB R7A 6A9, Canada; madisonb@brandonu.ca; 2 Department of Biology, Queen’s University, Kingston, ON K7L 3N6, Canadaelliot.gavrin@queensu.ca (E.G.); yuxiang.wang@queensu.ca (Y.W.); 3 World Wide Fund for Nature-China Shanghai Office, Suite 401, E Building, 1515 Zhongshanbei 2nd Road, Hongkou District, Shanghai 200092, China; wwren@tongji.edu.cn; 4 College of Environmental Science and Engineering, Tongji University, 1239 Siping Road, Yangpu District, Shanghai 202804, China

**Keywords:** biomanipulation, CHAB, cyanobacteria, microphytes, pseudofaeces, suspension filtration

## Abstract

Cyanobacteria harmful algal blooms (CHABs) are most commonly caused by the proliferation of the toxic species, *Microcystis aeruginosa*. It is therefore of considerable interest to develop biological control processes which are economically feasible and scalable for this cyanobacteria that produces the cyanotoxin, microcystin. Some gastropods that are abundant in freshwater ecosystems can filter feed on floating planktonic microphytes. We investigated this in the freshwater snail, *Sinotaia aeruginosa* which indiscriminately accumulated *M. aeruginosa*, *Chlorella vulgaris*, and *Trichormus variabilis* (syn. *Anabaena variabilis*) The initial filtration rates were approx. 44 and 19 mL · g_wwt_^−1^ · h^−1^ for unicellular and colony-forming *M. aeruginosa*, respectively. The pseudofaeces that were formed directly by filtration possessed a limited period of stability, and the bulk of the *M. aeruginosa* from pseudofaeces was eventually released back to the water column as undigested cyanobacteria. Nevertheless, the rate of sequestration of colonial *M. aeruginosa* into pseudofaeces was greater than its rate of release, thereby indicating that the temporary stability of pseudofaeces alone would be adequate to impede bloom formation. Therefore, these results provide evidence for using this gastropod in an effective preventative strategy for CHABs formation. Our results highlight the importance of understanding the impact of feeding mechanisms on ecosystem structure when proposing their use in biomanipulative processes aimed at correcting cyanobacteria impacted ecosystems.

## 1. Introduction

Cyanobacterial harmful algal blooms (CHABs) are high densities of these autotrophic prokaryotes in water bodies as a consequence of their rapid growth resulting in harm to other living organisms. The widespread global significance of CHABs on water security, ecosystems, and human health is already prevalent [1,2]. Eutrophication of aquatic ecosystems promotes the formation of rapid subsurface aggregations of cyanobacteria biomass that are toxin-forming, block sunlight from penetrating the water column, and deplete oxygen levels. The growth of these blooms will competitively persist until these enriched environments are depleted of nutrients, a scenario exacerbated by climate change [3,4,5,6,7].

Blooms may contain cyanobacteria from the genera *Microcystis*, *Dolichospermum*, *Aphanizomenon*, *Trichodesmium*, *Nodularia*, *Cylindrospermopsis*, and *Planktothrix*, most of which can produce cyanotoxins [8]. The predominant species, however, is *Microcystis aeruginosa* which produces the cyanotoxin, microcystin, that can result in significant harm to animals, humans, and ecosystems through foodweb interactions [9]. The levels of microcystin produced by *Microcystis* have been determined in several water bodies and shown to be associated with cell colony density at an estimated average amount of 0.5 µg cyanotoxin per µg Chl-*a* at moderate bloom levels [10,11,12,13] and the World Health Organization recommended value for recreational and swimming advisory for microcystins is 8 µg/L. This occurs when the cyanobacteria concentrations are over 20,000 cells/mL or 10–50 µg/L Chl-*a*.

Aquatic gastropod snails (i.e., *Viviparidae* family) are typically established in freshwater ecosystems affected by blooms. They enhance nutrient cycling by transforming organic detritus stored in the sediment into dissolved nutrients [14,15,16]. Importantly, many snails within related genera (e.g., *Bellamya*, *Sinotaia*, *Viviparus*) also continuously remove free-floating planktonic algae and cyanobacteria in a manner similar to bivalves that feed only through filtering biomass [17,18]. Most are regionally abundant and many are farmed locally for food, making them readily available for implementation in biological control of CHABs.

There are two mechanisms for consumption of microphyta by *Viviparidae*. Grazing involves taking in material through the mouth into the digestive system. Filtration uses a gill apparatus to encase suspended particles in pseudofaeces which may or may not be subsequently grazed. In the latter case, *Sinotaia aeruginosa* (syn. *Bellamya aeruginosa*) has been shown to significantly reduce phytoplankton biomass in the water column [19,20]. However, the varieties of phytoplankton removed by aquatic snails depend on species, as demonstrated by their impact on phytoplankton community composition. For example, cryptophytes dominate phytoplankton communities in the presence of *Pomacea canaliculata* [21], and colony-forming green algae are selectively removed by *S. aeruginosa* [22]. Even though *S. aeruginosa* appears to prefer green algae, it also ingests cyanobacteria, and it and other gastropods have been observed to have high tolerance and detoxification responses to microcystins [23,24,25]

The ability of *M. aeruginosa* to grow at an accelerated rate makes it one of the most prolific phototrophic microphytes in times of climate change [26,27]. Therefore, controlling eutrophication and consequential growth of this species are now crucial across changing global waterfronts [28,29]. Methods should strive to bolster existing ecological resources to support proactive and cost-effective bloom prevention solutions while naturalising and diversifying specific remediation strategies (e.g., biocontrol and biomanipulation). Ideally, proactive measures should be implemented that remove *Microcystis* from the water column before blooms become established [30]. Even temporary delays or interruptions of cellular aggregation would significantly aid downstream remediation efforts by reducing populations of offending cells.

Because reducing nutrient loading that causes eutrophication is difficult to achieve and resource expensive, proactive biological control strategies that remove the microphytes at a reasonable cost are attractive alternatives [8,31,32]. One such example would be the use of snail species to impede the colonial growth of cyanobacteria [33]. However, the degree of the effects of the filter-feeding processes in snails requires characterisation. In this paper, the removal of *Microcystis* as well as other microphytes by *S. aeruginosa* is investigated during the formation of pseudofaeces, the primary product of suspension filtration, and faeces produced by grazing. This study aims to evaluate this freshwater gastropod for effective biological control of CHABs.

## 2. Results

### 2.1. The Process of Suspension Filtration in Sinotaia Aeruginosa

When *S. aeruginosa* opens its operculum, water enters the inhalant syphon to the left of the head and passes posteriorly between gill filaments to the right side of the mantle cavity before exiting through the exhalant syphon. Suspended food particles caught on the gill filaments are mixed with mucus and carried into a food groove. These contents are rotated and compacted by ciliated epithelium into a cephalic food cord and moved towards the mouth, where they can be consumed.

This suspension filtration ability of *S. aeruginosa* was confirmed microscopically for all age and size classes, including newborn snails as small as 20 mg in size.

Unicellular and colony-forming *M. aeruginosa*, *Chlorella vulgaris*, and *Trichormus variabilis* were used to investigate the filtering ability of *S. aeruginosa* under controlled laboratory conditions. Microphytes were introduced into the inhalant syphon and the generated food cords, known as pseudofaeces, were collected prior to consumption. The process was very efficient because exhalant current samples did not contain any cells or colonies. The whole process of pseudofaeces production occurred within 3 min regardless of introduced microphyta (Table 1), indicating its non-selective nature. The snails could graze on all types of newly formed pseudofaeces by appropriately directing their snouts and radula. Subsequent faeces produced were released within 10 h (Table 1). Faeces produced from grazing on their own faeces are termed secondary faeces.

### 2.2. Structure and Decomposition of Pseudofaeces Produced

**Figure 1:**
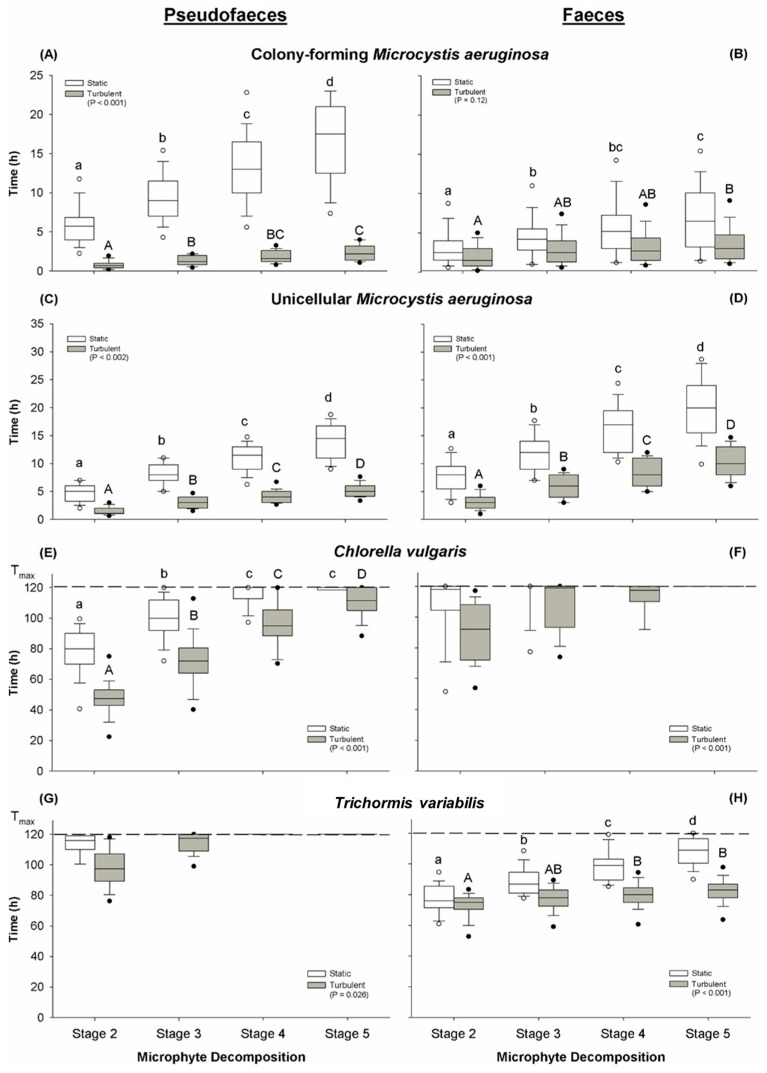
Decomposition time (hours) of *Sinotaia aeruginosa* pseudofaeces (left column of panels: **A**,**C**,**E**,**G**) and faeces (right column of panels: **B**,**C**,**F**,**H**) containing different microphyta: (**A**,**B**) colony-forming *Microcystis aeruginosa*; (**C**,**D**) unicellular *Microcystis aeruginosa*; (**E**,**F**) *Chlorella vulgaris*; (**G**,**H**) *Trichormus variabilis*. Boxes represent the time (in hours, box: median (line), 25th and 75th percentiles; outliers: 5th and 95th percentiles) taken for each microphyte pseudofaeces and faeces to reach the five stages of decomposition (Stages 1–5). Stage 1 (freshly produced) represents t = 0 and is assumed for each microphyte described. White boxes represent static conditions, and grey represents turbulent conditions. The maximum duration of the experiment was 120 h, represented by the horizontal hatched line in panels (**E**,**F**,**G**,**H**). Statistical comparisons were performed between the overall effect of static and turbulent conditions using ANOVA (*p* < 0.05) as indicated within the legend for each of the panels. Lettering denotes statistical differences (Tukey post hoc test, *p* < 0.05; *n* = 14) across the time to each stage for a particular condition; static (lower case) and turbulent (upper case).

Pseudofaeces were collected from immobilised snails as described in Section 5.3 below. Pseudofaeces containing intact microphytes were usually rope-shaped and heavier than water; however, those containing *Microcystis* became buoyant with increasing cyanobacterial content. The stages of decomposition (Table 2) were different among the microphyta under non-turbulent conditions (Figure 1, white boxes). The median times for bloom(colony)-forming *Microcystis* and unicellular *Microcystis* to decompose completely (Stage 5) were 17.0 h and 14.5 h, respectively (Figure 1A,C). Those pseudofaeces containing the *C. vulgaris* did not decompose completely (Stage 4 or Stage 5) by the end of the 120 h experimental period (Figure 1E). Decomposition of *T. variabilis* containing pseudofaeces reached Stage 2 with a median time of 114 h, with none of them entering Stage 3 by the end of the experimental period (Figure 1G). When turbulence was introduced to simulate natural conditions (grey boxes), the average time to decomposition for *M. aeruginosa* was reduced to approximately one third (Figure 1A,C). Pseudofaeces containing *C. vulgaris* also decomposed more rapidly (Figure 1E) but still required significantly longer time than *Microcystis*. *T. variabilis* still had very long decomposition times with turbulence and did not reach Stage 4 within 120 h (Figure 1G).

### 2.3. Quantifying Removal of M. aeruginosa via Suspension Filtration and Grazing

**Figure 2:**
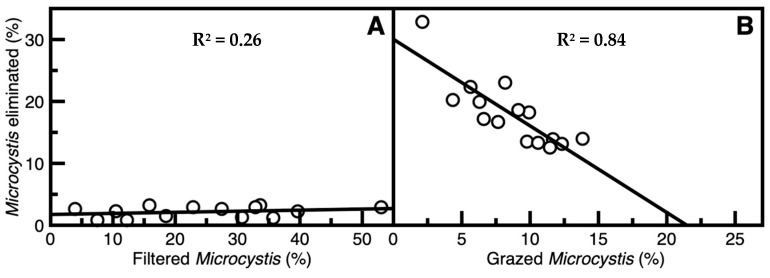
The proportion of removal of colony-forming *Microcystis aeruginosa* through (**A**) suspension filtration (y = 0.02x + 1.75), and (**B**) grazing (y = −1.40x + 30.0) by *Sinotaia aeruginosa*.

Free-roaming *S. aeruginosa* markedly reduced suspended *M. aeruginosa* concentrations as assessed by using the technique described in Section 5.3. Initial filtration rate (*F_R_*) of unicellular *M. aeruginosa* was 43.7 ± 5.9 mL g_wwt_^−1^_(snail)_ h^−1^ (mean ± SE, *n* = 14). However, for colony-forming *M. aeruginosa*, *F_R_* was much less at 19.0 ± 4.2. After a 4 h period, 24.6 ± 3.7% (mean ± SE, *n* = 14) of free colony-forming *M. aeruginosa* was removed from the water column. However, that eliminated through cell death, determined using chlorophyll estimates, possibly through mucus-related processes of suspension filtering, was only 2.1 ± 0.2% of the filtered *M. aeruginosa* (Figure 2A). The weakness of the relationship in Figure 2A, as indicated by the low R^2^ value, may be attributed to high variability at the lower limits of detection. Nevertheless, it is clear that all elimination (cell death) values are low. Analysis of the grazing *S. aeruginosa* showed that the proportional percentage of *M. aeruginosa* grazed (*G_M_*) was 8.3 ± 0.9% within 4 h, and the percentage of elimination (*E_g_*) was 18.0 ± 1.4% of the grazed cyanobacteria. Furthermore, with increases in grazing rate, elimination percentage decreased linearly (Figure 2B). These results indicate that there was minimal cyanobacterial elimination through both suspension filtration and grazing.

### 2.4. Structure and Decomposition of Faeces Produced

*Sinotaia aeruginosa* faeces obtained after the ingestion of pseudofaeces were composed of segments encapsulated in thick mucilage, with some contents being intact microphytes. As with pseudofaeces, faeces containing high amounts of unicellular or colony-forming *M. aeruginosa* floated. Rupturing of faeces under non-turbulent conditions occurred most rapidly for colony-forming *Microcystis* completing Stage 5 by 6.5 h (Figure 1B). With unicellular *Microcystis*, this was 20 h (Figure 1D). In the case of *Chlorella*, complete decomposition did not occur by 120 h (Figure 1F), and *Trichormus* required an average of 109 h to completely decompose (Figure 1H).

Decomposition time for *Microcystis* products was effectively halved during turbulent conditions (Figure 1B,D). On the other hand, turbulence had little or no effect on inducing decomposition in faeces containing *C. vulgaris* or *T. variabilis* (Figure 1F,H).

### 2.5. Structure and Decomposition of Secondary Faeces Produced

The structure of secondary faeces produced after ingestion of faeces in *S. aeruginosa* were similar to, but less dense than, the primary faeces, and decomposition times were significantly longer (*p* < 0.05, *n* = 50). Under non-turbulent conditions, none of the secondary faeces were structurally altered within 120 h. Those containing colony-forming *M. aeruginosa* exhibited some swelling, but maintained their original shape over 264 h. Even in turbulent conditions, only secondary faeces containing *Microcystis* had some minor breakage where the overall structure remained intact even after 90 h.

## 3. Discussion

### 3.1. Disassembly of Pseudofaeces and Faeces

It is important to characterise the stability of pseudofaeces and faeces of *S. aeruginosa* with respect to their environmental effects. This is a consequence of their microfloral content as well as their habitat. Our results showed significantly shorter decomposition times for both unicellular and colony-forming *M. aeruginosa* than for *T. variabilis* and *C. vulgaris* (Figure 1). Different physical properties, as well as structural and cellular configurations within these products, are likely to be the major factors influencing disassembly in aquatic environments. An important characteristic of *M. aeruginosa* is the presence of gas vesicles which appear to remain intact within the pseudofaeces and faeces. These increase their buoyancy while those containing the other types of microphyta sink. As such, the floating pseudofaeces and faeces containing *Microcystis* would be exposed to extra shear forces if surface turbulence occurred.

There was no general trend between pseudofaeces and faeces in the length of decomposition times within any microphyte type (Figure 1). However, in colony-forming *M. aeruginosa*, the major harmful bloom cyanobacterial form, the pseudofaeces in non-turbulent conditions decomposed much more slowly than faeces, whereas during turbulence this was rapid and not significantly different. This supports the utility of pseudofaeces production in biological control because of the prevalence of colony-forming *M. aeruginosa* during calm water conditions [34].

### 3.2. Removal and Elimination of Microcystis Through Ingestion by S. aeruginosa

Significant proportions of suspended *Microcystis* were removed from the water column through conversion into pseudofaeces that were grazed upon as a food source. However, there was low digestion of colony-forming *Microcystis* within pseudofaeces at less than 20%. Furthermore, *M. aeruginosa* elimination was inversely proportional to increased grazing (Figure 2). This in part could be explained by nutritional satiation [35], with recent evidence indicating that cyanobacteria are high-quality food resources for gastropods [36]. However, it is generally believed that higher nutritional value would reduce ingestion rate rather than digestion efficiency or the proportion of *Microcystis* eliminated by digestion. Further examination of faeces showed a high proportion of undigested *M. aeruginosa* still in colony form in some cases. Similar results were also found when colony-forming *M. aeruginosa* were consumed by filter-feeding silver carp (*Hypophthalmichthys molitrix*) whose digestive systems were also unable to fully eliminate this cyanobacterium [37]. This lack of digestive ability by silver carp had been confirmed through stable isotope analysis [19]. In contrast, it was noted that when *S. aeruginosa* consumed the filamentous cyanobacterium *T. variabilis*, or the unicellular chlorophyte *C. vulgaris*, microphyte survival was only 3% and 15%, respectively.

The low digestibility of *M. aeruginosa* may be due to the lack of certain enzymes in the digestive system required to break down mucilage. Many strains of *M. aeruginosa* have mucilage that is particularly robust in colony-forming variants [38]. This mucilage is mainly composed of microbial extracellular polymeric substances (EPS), primarily consisting of proteins, carbohydrates, and humic substances, among others [1]. Currently, there is no scientific literature reporting on the digestive enzymes of *S. aeruginosa* with respect to EPS, but [39] reported that lysozyme and cellulase enzymes are not present in silver (*H. molitrix*) or bighead carp (*H. nobilis*). Our data suggests that comparable enzymes may be absent in *S. aeruginosa*. Therefore, it does not appear that digestion of *M. aeruginosa* by *S. aeruginosa* could play a major role in the control of this cyanobacterium.

### 3.3. Filter-Feeding Snails in the Biological Control of M. aeruginosa

The present findings indicate that *M. aeruginosa* is efficiently encased in pseudofaeces through filter feeding by *S. aeruginosa*. The temporary nature of this encasement and the apparent ineffectiveness of this snail to digest the cyanobacteria may be drawbacks to its utility in biological control of CHABs. However, the rate of encasement is greater than that of the release of cyanobacteria from pseudofaeces such that it could significantly delay CHABs development. This process can be expressed in the following system model.

In the presence of *S. aeruginosa*, *M. aeruginosa* is encapsulated in pseudofaeces. Given that *F_r_* is the suspension filtration rate per unit fresh weight of snail and *T_r_* is the time for the release of *M. aeruginosa* through pseudofaeces rupturing, then *V_max_*, which is the maximum volume of managed water in the system, is defined as

(1) $$V_{max}=F_{r}\times T_{r}$$

The ability of snails to remove suspended *M. aeruginosa* from the water column is quantified as *F_s_*, the specific filtration rate defined as the proportion of a given water volume cleared of *M. aeruginosa* per unit time.

(2) $$F_{s}=\frac{F_{r}\;}{V_{max}}$$

The *d*(*t*), exponential decay function, represents the progressive removal of cyanobacterial biomass Via filtration from *M*_0_, the initial concentration of *M. aeruginosa*, and *r*(*t*), the release function, represents the delayed reintroduction of biomass from degrading pseudofaeces. These functions express the concentration of *M. aeruginosa* remaining in suspension at time (*t*), per gram of snail biomass.

The decay rate is expressed as an exponential decay function.

(3) $$d\left(t\right)=M_{0}\cdot {\left(1-F_{s}\right)}^{t}$$

At time (*t*), a proportion of the original *M. aeruginosa* biomass is returned to the water column as pseudofaeces degrade once a fixed release time *T_r_* has elapsed.

(4) $$r\left(t\right)=\left\{\begin{array}{r} 0,\;t<T_{r}\\ F_{s}\cdot (M_{0}\cdot {\left(1-F_{s}\right)}^{t-T_{r}}),\;t\geq T_{r} \end{array}\right.$$

Taking into account both filtration and the delayed return of pseudofaeces, *M*(*t*), the net concentration of *M. aeruginosa* in the water column at hour *t* is:

(5) $$M(t)=d\left(t\right)+r(t)$$

(6) $$M\left(t\right)=M_{0}\cdot {\left(1-F_{s}\right)}^{t}+\{t<T_{r}\;:0,\;F_{s}\cdot (M_{0}\cdot {\left(1-F_{s}\right)}^{t-T_{r}})\}$$

In the context of the proposed organism system assessed in the present study, one gram of wet-weight *S. aeruginosa* biomass can be processed at the rate of *F_r_* at approximately 19 mL of water per hour by converting suspended *M. aeruginosa* into pseudofaeces. These pseudofaeces degrade after 17 h (*T_r_*), releasing the *M. aeruginosa* back into the environment. Therefore, a single gram of *S. aeruginosa* can sustain improved water quality up to 323 mL (*V_max_*) of water at a specific filtration rate (*F_s_*) of ~6% of the total volume per hour. In 11.4 h, 50% of the *Microcystis* concentration would be contained in pseudofaeces, 75% at 25.4 h, and 95% at 52.0 h. This high proportion of encasement of the cyanobacteria would restrict growth and hence contribute to the curtailment of CHAB formation.

Additionally, the resolution of other confounding issues could further enhance the CHAB control effectiveness of this filter-feeding snail in natural habitats. One of these is its limited ability to filter floating cyanobacteria because it inhabits the substrate, although this includes the shoreline. This could be overcome through its deployment when the organisms are in their unicellular form prior to becoming floating colonies [40]. Another concern is that *M. aeruginosa* passes through the snail with relatively low digestion, as appropriate gut enzymes appear to be absent. Nevertheless, continuous filtration into pseudofaeces by *S. aeruginosa* would be expected to significantly delay development of CHABs.

The other benefits of employing *S. aeruginosa* in the biological control of cyanobacterial algal blooms are numerous. They can multiply quickly and live at high densities [41,42,43], are easily reared in captivity [41] such that up-scaling to waterbody size could be readily accomplished, and tolerate cyanotoxins [40]. Another advantage is that filter-feeding snails are found throughout the globe [20,44,45,46]. For example, Olden and colleagues [20] suspected that *Bellamya chinensis* may display an ontogenetic shift in feeding behaviour from primarily radula grazing to filter feeding with increasing size. The present study directly observed suspension-feeding behaviour in all age groups of *S. aeruginosa*. A similar process was also observed in *Viviparus georgianus* native to the St. Lawrence River in Canada (unpublished data), further supporting that this might be a common property of numerous gill-breathing freshwater gastropods.

## 4. Conclusions

The mechanism of suspension filtration in *S. aeruginosa* captures *M. aeruginosa* through encapsulation in pseudofaeces. Though the stability of pseudofaeces is transitory, the process would effectively render the encased microphytes unable to interact with the aquatic environment. In addition, some degree of digestion and stability of faeces and secondary faeces would also contribute to the overall process of biological control. At times when blooming conditions are ideal in an eutrophic aquatic environment, delaying colony-forming *Microcystis* from growing to a critical biomass could substantially impede the formation of hazardous CHABs, especially when there is adequate early detection [34]. It is this premise by which we suggest that the suspension filtration mechanism of *S. aeruginosa*, and likely that of similar freshwater snails, could be exploited in greater numbers for the proactive control of *M. aeruginosa*.

## 5. Materials and Methods

### 5.1. Collection and Maintenance of Sinotaia aeruginosa

*Sinotaia aeruginosa* (syn. *Bellamya aeruginosa*) snails were collected from subtropical Dianshan Lake (31.093963° N, 120.985338° E), Shanghai, China, and held in 200 L aquaria equipped with aeration and biological filters at 24 °C. Illumination at 125 μmol m^−2^ s^−1^ by cool-white fluorescent lighting provided a 14:10 h light-dark cycle. Except where indicated differently, the snails used weighed 2.6 ± 0.22 g_wwt_ (mean ± SD, *n* = 100) and were fed a diet of *Chlorella vulgaris* (Chlorophyta) of approximately 16 mg snail^−1^·d^−1^.

### 5.2. Cyanobacterial and Algal Species Collection and Culture

Four types of microphyta presented in Table 3 were cultured in laboratory conditions for this study. Unicellular *Chlorella vulgaris* and *Microcystis aeruginosa* were cultured in BG11 medium [47]. Colony-forming *M. aeruginosa* had an initial average colony size based on the greatest axial diameter of 945 ± 399 µm (mean ± SD, *n* = 100). After their culture in the MA medium [48] for one month, the colony size was reduced to 583 ± 335 µm (*n* = 100). The chain-shaped colony-forming cyanobacterium, *Trichormus variabilis*, was similarly cultured in BG11. Serial stock cultures grown in duplicate using autoclaved nutrient media were used to inoculate 1 L cultures in Erlenmeyer flasks maintained in a plant culture environmental chamber (Leigu SPX-80-II, Shanghai, China) at 24 °C in a 14:10 h light/dark cycle with illumination of 25 μmol·m^−2^ s^−1^ without shaking.

### 5.3. Microscopic Characterisation of Suspension Filtering Process

To observe the suspension-feeding process of *S. aeruginosa,* a setup was used that prevented snails from grazing to permit visual assessment of the filtering process. This comprised a microcentrifuge tube (Diamed Inc., Mississauga, ON, Canada) fixed to the side of a 100 mL transparent plastic box (MilliporeSigma Ltd., Oakville, ON, Canada) using low-temperature melting glue. Each snail was similarly secured to the tube by its shell such that its head–foot aperture faced outwards and was free of obstruction. Suspension-feeding processes were observed using a dissecting microscope (Nikon SMZ800, Tokyo, Japan) and recorded using an iPhone 6 camera under LED lighting (IKEA Jansjo, Stockholm, Sweden). Filtering behaviour appeared to proceed normally after attachment to the experimental apparatus.

Experiments were performed in this manner across age classes from newly born (~0.02 g_wwt_) to 4 y old individuals (~5.5 g_wwt_). Single snails were placed in 1 L beakers of aquarium water and deprived of food for 48 h with continuous removal of faeces until defaecation ceased. Snails were then permitted to acclimate for 30 min in the attached manner described above. One-millilitre syringes filled with either unicellular or colonial cyanobacteria or algae were dispensed near the syphon intake region of each snail. The resultant pseudofaeces were then collected from the filter output region using a 250 µL Hamilton syringe and assessed microscopically.

### 5.4. Filtration Rate and Removal of M. aeruginosa Through Suspension Filtration and Grazing of Mobile Snails

Removal of microphytes in the context of this paper is defined as being taken out of the water column, whereas elimination occurs through cell death. The latter is measured by the loss of Chl-a signal through cell lysis (see Section 5.6 below). To quantify the activity of the suspension filtering process of freely mobile snails, measurements were performed in chambers containing 300 mL of filtered lake water. To remove any possible adhering microphytes, shells of *S. aeruginosa* were gently but thoroughly cleaned with a soft brush 24 h prior to each experiment. Three snails of approximately 2.6 g_wwt_ each were used per experiment, and microphytes were suspended in the water at an initial Chl-*a* concentration of 150 µg L^−1^. This represents conditions in which CHABs occur [13] at approximately 1 × 10^5^ cells/mL for *M. aeruginosa*. Newly formed pseudofaeces were continuously collected over 4 h using Hamilton syringes (MilliporeSigma Ltd., Oakville, ON, Canada) to prevent pseudofaeces from being subsequently grazed. Then, water samples were taken from the experimental chambers to quantify the loss of free *M. aeruginosa*. Chl-*a* concentration was measured in tank water and in pseudofaeces samples to quantify microphyte amounts. The rate of filtration (*F_R_*) was the volume that passes through the filtering apparatus over a given interval [49,50], measured through the removal of Chl-*a* per unit time [51]:

(7) $$F_{R}=\frac{ln{(C}_{i})-{ln(C}_{f})}{t}\times \frac{V}{W}$$

where *F_R_* was the rate of filtration (mL·g_wwt_^−1^ h^−1^), *V* was the volume of water in the tank (L), *W* was the wet weight (g_wwt_) of snails per tank, *C_i_* was the initial and *C_f_* was the final Chl-*a* concentration in the experiment (µg L^−1^), and *t* was the total duration of the experiment (h). The proportion (%) of filtered microphyte (*F_M_*) was determined using the following formula:

(8) $$F_{M}=(\frac{C_{f}\times V_{f}}{C_{i}\times V_{i}})\times 100\%$$

where *V_i_* was the initial volume (L), and *V_f_* was the final volume of water in the system. Collecting pseudofaeces necessitates water removal that changes the total volume.

Direct measurement of the proportion of *M. aeruginosa* sequestered in pseudofaeces produced through suspension filtration (*C_s_*) was

(9) $$C_{s}=(\frac{C_{ps}\times V_{ps}}{{(C}_{i}\times V_{i})-(C_{f}\times V_{f})})\times 100\%$$

where *C_ps_* was the Chl-*a* concentration of collected pseudofaeces (µg L^−1^) and *V_ps_* was the volume of collected pseudofaeces (L).

To determine the effective elimination of microphytes through *S. aeruginosa’s* digestive process, an experimental apparatus was employed that prevented secondary ingestion, i.e., grazing of their own faeces. Two tubes were used in sequence during the experiment. One contained a nylon netting (5 mm mesh was large enough to permit pseudofaeces and faeces to pass through) barrier set one third from the floor (tube A). This partition restricted snail movement to the bottom of the tube while permitting it to directly graze on floating *M. aeruginosa* as well as suspension feed. The second tube had two nylon partitions set at one fifth the distance from both top and bottom (tube B). Snails were placed in between these nylon partitions to prevent access to faeces containing *M. aeruginosa* that can sink or float. Each tube contained 200 mL of filtered lake water. Colony-forming *M. aeruginosa* (Chl-*a* concentration at 150 µg L^−1^, equivalent to 3.5 × 10^6^ cells mL^−1^) were added to tube A with one 2.6 g snail. After 4 h in tube A, *S. aeruginosa* were removed and placed in tube B for an additional 12 h to collect the faeces produced. Faeces were removed every hour from tube B and transferred to clean 15 mL centrifuge tubes for immediate analyses. Moreover, 5 mL water samples were taken at the beginning and end of the initial 4 h exposure in tube A in order to estimate the concentration of the remaining free cyanobacteria.

Similarly to the suspension experiments, the percentage of grazed *M. aeruginosa* (*G_M_*) was calculated as follows:

(10) $$G_{M}=(\frac{C_{f-a}\times V_{f-a}}{C_{i-a}\times V_{i-a}})\times 100\%$$

where *C_i−a_* is the initial Chl-*a* concentration (µg L^−1^), *C_f−a_* was the final Chl-*a* concentration in tube A, *V_i−a_* was the initial volume (L), and *V_f−a_* was the final volume of the water in tube A. Collecting faeces during the experiment removes a certain amount of water.

Proportion (%) of *M. aeruginosa* elimination (cell death) through grazing (*E_g_*) was calculated through the formula:

(11) $$E_{g}=(\frac{\left(C_{f}\times V_{f})+{(C}_{f-b}\times V_{f-b}\right)}{{(C}_{i-a}\times V_{i-a})-(C_{f-a}\times V_{f-a})})\times 100\%$$

where *C_f_* was the Chl-*a* concentration (µg L^−1^), *V_f_* was the volume of collected faeces, *C_f−b_* was the final Chl-*a* concentration (µg L^−1^), and *V_f−b_* was the final volume of the water in tube B (L).

### 5.5. Decomposition Time of Pseudofaeces and Faeces

**Figure 3:**
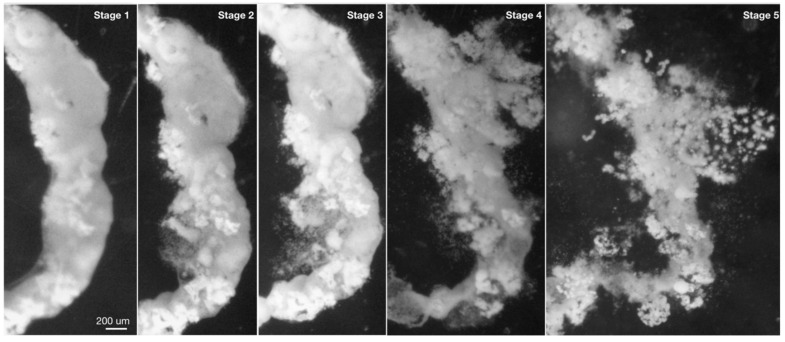
Photographs of pseudofaeces of *Sinotaia aeruginosa* containing colony-forming *Microcystis aeruginosa*. Stages 1–5 are described in Table 2.

**Figure 4:**
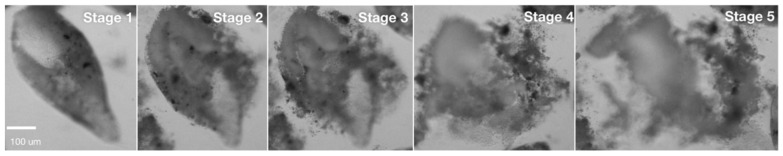
Photographs of faeces of *Sinotaia aeruginosa* containing colony-forming *Microcystis aeruginosa*. Stages 1–5 are described in Table 4.

The decomposition times of pseudofaeces containing different cyanobacteria and algae were examined under both non-turbulent and turbulent conditions. Fourteen snails, each weighing 2.6 ± 0.22 g, were placed into 1 L beakers where they were deprived of food for 48 h and their faeces removed until defecation ceased. Snails were then permitted to filter feed, and intact pseudofaeces were then gently collected and submerged in MA medium in individual wells of a 12-well strip plate (MilliporeSigma Ltd., Oakville, ON, Canada) and immediately examined microscopically. Plates were sealed to prevent evaporation during incubation with or without orbital shaking at 60 RPM, used to simulate the turbulent conditions of natural environments. The pseudofaeces samples were examined to record decomposition status every 20 min for the first 6 h, then every hour during the day and every 2 h during the night for a total of 120 h. Table 2 provides definitions for, and Figure 3 presents sample pictures of each decomposition stage. Experiments were also similarly performed to determine faeces decomposition based on the stages described in Table 4 and shown in Figure 4. For reference, a comparison of the characteristics of pseudofaeces and faeces produced by *S. aeruginosa* is presented in Table 5.

### 5.6. Sample Processing and Chlorophyll-A Measurement

Chlorophyll-*a* (Chl-*a*) measurement was performed following a standard spectrophotometric method, protocol ISO 10260:1992/SL88-2012 [52]. Briefly, water samples were pre-filtered with Whatman GF/F Glass Microfiber Filters (Whatman, United Kingdom) prior to the measurement of Chl-*a* as a proxy quantification for microphyte concentration in solution. The protocol lyses cells and stops all metabolic activity through cold acetone extraction followed by specific Chl-*a* spectrometric determination.

### 5.7. Optics and Photography

Photographs and microscopic images were taken using time-lapse digital video recording under LED illumination.

### 5.8. Statistical Analysis

Statistical comparisons were performed on data using regression and analysis of variance (ANOVA) complemented by Tukey Post hoc analyses (*p* < 0.05) in R version 3.2.1, and plotted using Datagraph 4.1 (Visual Data Tools, Inc., Chapel Hill, NC, USA) or Sigmaplot 11 (Grafiti LLC., Palo Alto, CA, USA).

## Figures and Tables

**Table 1:** Duration of time to release pseudofaeces from suspension filtration and faeces from grazing by *Sinotaia aeruginosa* on different cyanobacteria and algae. All food types tested were observed to be filterable despite the variation in size. No residual cyanobacteria or algae were observed in the exhalant current for any of the treatments with the four food types. Values given are mean ± SE (*n* = 25).

			*Cyanobacteria*		*Chlorophyta*
Process	Time to Release	*Microcystis aeruginosa* (Unicellular)	*Microcystis aeruginosa* (Colony-Forming)	*Trichormus variabilis* (Colony-Forming)	*Chlorella vulgaris* (Unicellular)
Suspension filtering	pseudofaeces (s)	131 ± 9.2	112 ± 13.6	141 ± 10	125 ± 5.8
Grazing	faeces (h)	8.3 ± 0.7	9.1 ± 2.3	8.9 ± 0.8	9.4 ± 0.6

**Table 2:** Classification of pseudofaeces in *Sinotaia aeruginosa* based on morphological traits and degree of decomposition.

Stage	Shape	Surface	Content Release	Notes
1	Tight rope-shaped	Smooth and constricting content	None	Original status
2	Rope- shaped, but inflated	Smooth with loose content	Few cells	
3	Inflated, slight loss of original shape	Loose with coarse edges	Numerous cells	
4	Deformed, major loss of integrity	Unrestricting with coarse edges	Majority of cells	
5	Deformed, complete loss of integrity	No clear edges	All cells	Completely decomposed

Representative images of each stage are presented in Section 5.2.

**Table 3:** Summary of cyanobacteria and algae employed in this study including cellular structure, size and predominant distribution in natural habitat water column.

Taxon	*Cyanobacteria*			*Chlorophyta*
Species:	*Microcystis aeruginosa*	*Microcystis aeruginosa*	*Trichormus variabilis*	*Chlorella vulgaris*
Strain ID:	† FACHB-905	Collected from Dianshan Lake, China	Collected from Dianshan Lake, China	† FACHB-8
Cellular structure:	Unicellular	Irregularly shaped colony-forming	Filamentous colony- forming	Unicellular
Cell size *: (µm)	3–5	4–7	5–8	3–5
Colony size *: (µm)	N/A	100–500	60–250	N/A
External mucilage:	No	Yes	No	No
Distribution in water column:	Suspended uniformly throughout	Buoyant, floats to surface	Floats to surface, may attach to substrate	Sinks to benthic zone, suspended by turbulence

* Cell and colony sizes were measured at greatest axial linear dimensions. † Freshwater Algae Culture Collection at the Institute of Hydrobiology (FACHB), Wuhan, China.

**Table 4:** Classification of decomposition states of *Sinotaia aeruginosa* faeces based on morphological form and degree of breakdown.

Stage	Shape	Surface	Content Release	Notes
1	Fusiform shape, connected through mucilage	Smooth and constricting content	None	Original status
2	No change	Smooth; small rupture points	Few cells	
3	Slight loss of original shape	Edge discernible; Multiple rupture points	Numerous cells	
4	Deformed, major loss of integrity	Most barrier missing	Majority of cells	
5	Deformed, complete loss of integrity	No clear edges	All cells	Completely decomposed

Representative images of each stage are presented in Figure 4.

**Table 5:** Comparison of characteristics between pseudofaeces and faeces produced by *Sinotaia aeruginosa.*

Characteristics	Pseudofaeces	Faeces	Secondary Ingestion Faeces
Shape	Rope-shaped	Fusiform	Fusiform
Configuration	Trapped suspended particles with mucilage	Thick mucilage outside	Thick mucilage outside & mucilage inside
Hardness	Soft	Firm	Firm
Colour	Colour of contents	Colour of contents	Colour of contents

## Data Availability

The original contributions presented in this study are included in the article. Further inquiries can be directed to the corresponding author.
